# CPSF4 promotes triple negative breast cancer metastasis by upregulating MDM4

**DOI:** 10.1038/s41392-021-00565-9

**Published:** 2021-05-19

**Authors:** Kaping Lee, Qiufan Zheng, Qianyi Lu, Fei Xu, Ge Qin, Qinglian Zhai, Ruoxi Hong, Miao Chen, Wuguo Deng, Shusen Wang

**Affiliations:** 1 Sun Yat-sen University Cancer Center, State Key Laboratory of Oncology in South China, Collaborative Innovation Center for Cancer Medicine, Guangzhou, China; 2 The Sixth Affiliated Hospital of Sun Yat-Sen University, Guangzhou, China

**Keywords:** Breast cancer, Oncogenes

**Dear Editor,**

Breast cancer (BrC) is the most common cancer in women. Triple negative BrC (TNBC) is the subtype with highly aggressive clinical behaviors and heterogeneity. Metastasis is the leading cause of TNBC-related deaths, but its mechanism is not well-understood. Apart from PIK3CA, TP53, and PTEN, few recurrent mutations have been identified in TNBC so far, suggesting that TNBC phenotype could be driven by nongenetic alterations such as aberrant expression of oncogenes. Cleavage and polyadenylation-specific factor complexes (CPSFs) participate in the processes of transcription initiation, cleavage, and formation of the poly(A) tail, as well as RNA splicing^1^. Among the CPSF proteins, CPSF4 has been reported to cause the malignant phenotypes in lung cancer by upregulating the transcription of telomerase reverse transcriptase^2^. However, the role of CPSF4 in TNBC remains unclear. Here we aimed to investigate the potential role of CPSF4 in TNBC metastasis and the underlying mechanism.

First, we analyzed the clinical significance of CPSF4 using GenExMiner and The Cancer Genome Atlas (TCGA). Elevated mRNA level of CPSF4 was associated with shorter overall survival (OS) in BrC patients, and the expression of CPSF4 was significantly higher in basal-like BrC than that in normal breast tissues and other BrC subtypes (Supplementary Fig. S1a, b). Consistently, analysis of TCGA data showed that CPSF4 mRNA level was higher in basal-like and HR + BrCs than normal breast tissues (Supplementary Fig. S1c–e). These results indicate that CPSF4 may play a critical role in TNBC. We then detected the protein abundance of CPSF4 in BrC tissues and cell lines. The expression of CPSF4 was higher in BrC compared to that in normal breast tissues and TNBC cells had higher expression of CPSF4 than cells in other subtypes (Supplementary Fig. S1f, g). Accordingly, we performed subsequent experiments in MDA-MB-231 and SUM-159PT TNBC cell lines. Transwell migration and matrigel invasion assays were performed after transfecting cells with small interfering RNA/short hairpin RNA to suppress the expression of CPSF4. As shown in Fig. 1a and Supplementary Fig. S2a, knockdown of CPSF4 significantly decreased the capacity of cell invasion and migration. In contrast, transwell migration and matrigel invasion assays performed in cells with lentiviral vector-mediated overexpression of CPSF4 showed that increased expression of CPSF4 significantly enhanced the invasion and migration in both cell lines (Fig. 1b and Supplementary Fig. S2b). Epithelial–mesenchymal transition (EMT) is one of the most important steps in metastasis. To verify whether CPSF4 induces EMT, we detected the expression of Snail, ZEB1, and vimentin in response to changes of CPSF4 expression. Our results demonstrated that knockdown of CPSF4 inhibited EMT, whereas the expressions of EMT-related markers were increased after overexpression of CPSF4 (Supplementary Fig. S2c, d), proving that CPSF4 promoted TNBC cells metastasis through inducing EMT. Moreover, we observed that cell proliferation was repressed by CPSF4 knockdown in TNBC cells (Supplementary Fig. S2e, f).

**Fig. 1:**
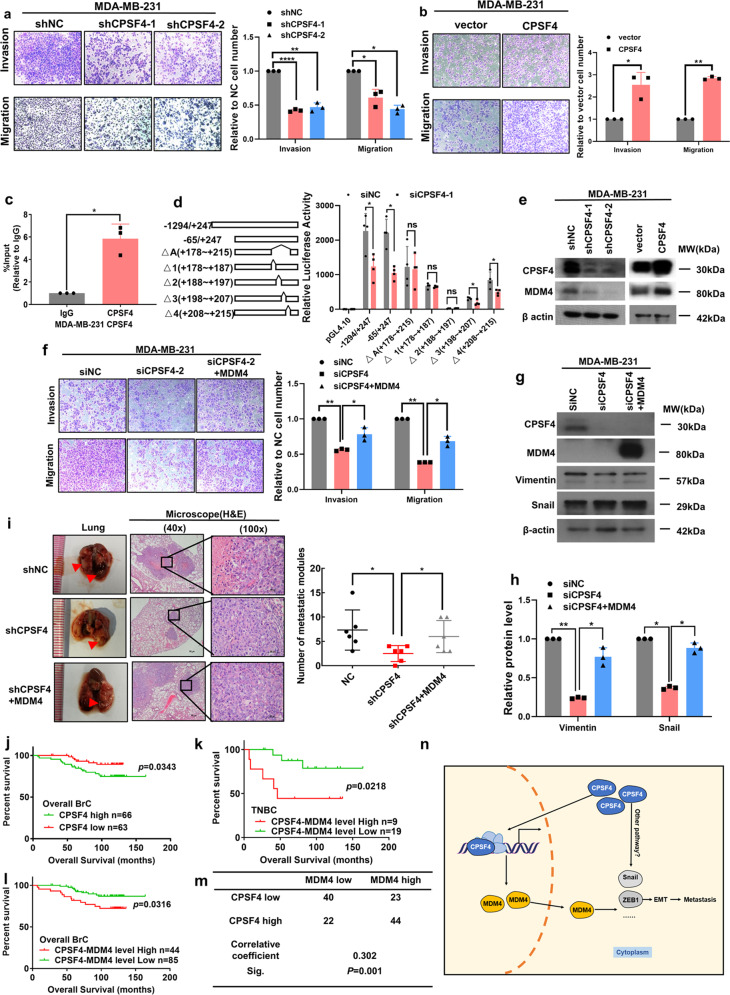
CPSF4 promotes triple negative breast cancer metastasis by upregulating MDM4. **a**, **b** Both MDA-MB-231 and SUM-159PT TNBC cells were transfected with CPSF4 shRNA and overexpression lentivirus, respectively. The cells above were used to perform transwell migration and matrigel invasion assays, respectively. **c** ChIP-qPCR was performed with CPSF4-specific antibody. **d** The promoter region of MDM4 was cloned into the pGL4.10 vector, including a 1541 bp fragment (−1294 ~ +247 to TSS), a 312 bp fragment (−65 ~ +247 to TSS) from the MDM4 promoter. Moreover, MDM4 promoter luciferase reporter plasmids, which deleted △A (a possible binding region of CPSF4 identified by ChIP-seq, +178 ~ +215 to TSS), △1 (+178 ~ +187 to TSS), △2 (+188 ~ +197 to TSS), △3 (+198 ~ +207 to TSS), and △4 (+208 ~ +215 to TSS) regions were conducted, respectively. Dual-luciferase reporter assay was performed to detect MDM4 promoter activity after silencing CPSF4. **e** Western blotting was performed to detect the expression of MDM4 after silencing or overexpressing CPSF4, respectively. **f** MDA-MB-231 cells were transfected with siRNA-CPSF4 or MDM4-overexpressing lentivirus. The cells above were used to perform transwell migration and matrigel invasion rescue assays, respectively. **g** Expression of EMT-related markers were detected by western blotting. **h** Band intensity of western blotting. **i** MDA-MB-231 cells were transfected with shRNA-CPSF4 or MDM4-overexpressing lentivirus. These cells were injected into the tail vein of 4-week-old nude mice to establish metastatic models (*n* = 6). Metastatic nodules were analyzed by the H&E staining and the numbers of nodules were counted. **j** The Kaplan–Meier plotter survival analysis was performed to analyze the overall survivals related to high or low expression of CPSF4 in overall BrC patients (*P* = 0.0343). **k**, **l** The Kaplan–Meier plotter survival analysis was performed to analyze the overall survivals related to high or low expression of CPSF4-MDM4 level in TNBC (*P* = 0.0218) and overall BrC patients (*P* = 0.0316), respectively. **m** The *χ*^2^-correlation analysis of CPSF4 and MDM4. **n** The diagram of this study. The results were presented as mean ± SD from three independent trials, *P < 0.05, **P < 0.01, ***P < 0.001, ****P < 0.0001. TSS transcription start sites.

Next, we performed chromatin immunoprecipitation (ChIP)-sequencing to identify the target genes of CPSF4, which was found to correlate with the promoter regions of 65 genes out of 162 candidates. KEGG (Kyoto Encyclopedia of Genes and Genomes) pathway analysis revealed the target genes were related to genome nucleotide-excision repair, epidermal growth factor receptor signaling, and metastasis (Supplementary Fig. S3a, b). We examined the expression of the metastasis-related genes by quantitative reverse transcription PCR (qRT-PCR) and discovered that the expression of Mouse double minute 4 (MDM4), a suppressor of p53^3^, decreased markedly after CPSF4 knockdown (Supplementary Fig. S3c, d). We then confirmed the binding of CPSF4 at the MDM4 gene promoter by ChIP-quantitative PCR (Fig. 1c). In addition, we constructed MDM4 promoter-driven luciferase reporter plasmids, which contained the full-length MDM4 promoter or its different deletion fragments. As shown in Fig. 1d, the +178 ~ +215 region was crucial to the transcription activation of MDM4 and the +188 ~ +197 region contained the essential elements for promoter activity, whereas the +178 ~ +187 region was indispensable to CPSF4-dependent transcription. In line with the observations above, western blotting showed that CPSF4 promoted the expression of MDM4 (Fig. 1e and Supplementary Fig. S3f). Given that CPSF4 had no DNA-binding domain, we surmised that CPSF4 was recruited to the MDM4 promoter by other proteins to enhance MDM4 transcription. It is known that transcriptional regulation can be coupled to RNA splicing and CPSF4 has been reported to participate in the alternative splicing of TP53 mRNA^4^. Therefore, we performed qRT-PCR to determine whether CPSF4 functions in the alternative splicing of MDM4-S, the variant that lacks exon 6 of the full-length transcript and is abundant in proliferating cell^5^. As shown in Supplementary Fig. S4a, b, the level of MDM4-S remained unaffected despite changed CPSF4 expression. Moreover, we tested the stability of MDM4 mRNA and protein, and found CPSF4 had no effect on it (Supplementary Fig. S4c, d). Also, neither knockdown nor overexpression of CPSF4 influenced the expression of p53 and MDM2 (Supplementary Fig. S4e), the interaction partners of MDM4. Collectively, these findings suggest that CPSF4 transcriptionally regulated the expression of MDM4.

Furthermore, we performed rescue assays to determine whether CPSF4 promoted metastasis of TNBC cells through MDM4. Transwell migration, matrigel invasion, and wound scratch assays unanimously showed that knockdown of CPSF4 inhibited TNBC cell migration and invasion, whereas overexpression of MDM4 partially reversed the effects caused by CPSF4 silencing (Fig. 1f and Supplementary Fig. S5a, c, d). Consistently, knockdown of CPSF4 decreased the expression of Snail and vimentin, which was rescued partly by MDM4 overexpression (Fig. 1g, h and Supplementary Fig. S5b). Afterward, we injected TNBC cells into the tail vein of 4-week-old nude mice to establish in vivo metastatic model. Twelve weeks later, we separated the lungs from mice and counted the number of metastatic nodules. As shown in Fig. 1i, knockdown of CPSF4 reduced the number and size of metastatic nodules compared with the control group, whereas MDM4 overexpression reversed these effects. Immunohistochemical (IHC) staining showed the expression of vimentin and ZEB1 diminished after CPSF4 silencing, and such effects were abolished by overexpressing MDM4 (Supplementary Fig. S6a). These findings demonstrate that CPSF4 regulates TNBC cell metastasis through MDM4 in part.

Finally, to clarify the association between CPSF4/MDM4 expression and BrC outcome, we used IHC staining to examine the expression of CPSF4 and MDM4 in 129 BrC patients, including 101 non-TNBC and 28 TNBC patients. Kaplan–Meier analysis showed that high expression of CPSF4 was correlated with poor OS in both TNBC (*P* = 0.3960) and overall BrC patients (*P* = 0.0343), although the correlation was not statistically significant in TNBC group due to small sample size (Fig. 1j and Supplementary Fig. S6b). Further, Kaplan–Meier analysis showed that high CPSF4-MDM4 level was associated with worse OS in TNBC and overall BrC populations (*P* = 0.0218 in TNBC, *P* = 0.0316 in overall BrC) (Fig. 1k, l). In addition, multivariate Cox proportional hazards regression analysis showed that CPSF4-MDM4 level was an independent prognostic factor (Supplementary Tables S1 and 2). We also calculated the concordance index (C-index) to evaluate the accuracy for predicting survival. The result showed that the C-index for OS prediction in the model including CPSF4-MDM4 level (0.833, 95% confidence interval (95% CI) = 0.647–1.02) was higher than that excluding CPSF4-MDM4 level (0.726, 95% CI = 0.504–0.948) (Supplementary Table S3). Correlation analysis showed that the expression of MDM4 was positively correlated with the expression of CPSF4 (correlation coefficient = 0.302, *P* = 0.001) (Fig. 1m).

To our knowledge, our study is the first to demonstrate that CPSF4 promotes TNBC metastasis by upregulating MDM4 and inducing EMT. Mechanistically, we discovered that CPSF4 transcriptionally regulated MDM4 but not its alternative splicing, mRNA, or protein stability. Our findings also revealed that patients with high CPSF4-MDM4 level were related to worse prognosis in TNBC, which makes them potential prognostic biomarkers and therapeutic targets of TNBC.

## Supplementary information

supplementary data

supplementary materials ChIP-seq data

## Data Availability

The ChIP-seq data of CPSF4 in MDA-MB-231 cells during this study are included in this published article and its Supplementary Information files.

## References

[1] Li YZ (2001). The 3′-end-processing factor CPSF is required for the splicing of single-intron pre-mRNAs in vivo. RNA.

[2] Chen W (2014). CPSF4 activates telomerase reverse transcriptase and predicts poor prognosis in human lung adenocarcinomas. Mol. Oncol..

[3] Shvarts A (1996). MDMX: a novel p53-binding protein with some functional properties of MDM2. EMBO J..

[4] Dubois J (2019). The non-structural NS1 protein of influenza viruses modulates TP53 splicing through the host factor CPSF4. J. Virol..

[5] Rallapalli R (1999). A novel MDMX transcript expressed in a variety of transformed cell lines encodes a truncated protein with potent p53 repressive activity. J. Biol. Chem..

