# Supplementary material for: CPSF4 promotes triple negative breast cancer metastasis by upregulating MDM4
Source: Signal Transduct Target Ther. 2021 May 19;6:184. doi: 10.1038/s41392-021-00565-9 (PMC8131696; doi:10.1038/s41392-021-00565-9)
Supplement: Supplementary file 1 — supplementary data [file 41392_2021_565_MOESM1_ESM.docx]

Supplementary Materials for

CPSF4 promotes triple negative breast cancer metastasis by upregulating MDM4

Kaping Lee^1,§^, Qiufan Zheng^1,§^, Qianyi Lu^1,§^, Fei Xu^1,§^, Ge Qin^2^, Qinglian Zhai^1^, Ruoxi Hong^1^, Miao Chen^1,*^, Wuguo Deng^1,*^, Shusen Wang^1,*^

1. Sun Yat-sen University Cancer Center; State Key Laboratory of Oncology in South China; Collaborative Innovation Center for Cancer Medicine, Guangzhou, China;

2. The Sixth Affiliated Hospital of Sun Yat-Sen University, Guangzhou, China.

§These authors contributed equally to this work.

*Correspondence to:

Shusen Wang, Sun Yat-sen University Cancer Center, Guangzhou, China, E-mail: [wangshs@sysucc.org.cn](mailto:wangshs@sysucc.org.cn), Tel : 86-20-87342491;

Wuguo Deng, Sun Yat-sen University Cancer Center, Guangzhou, China, E-mail: [dengwg@sysucc.org.cn](mailto:wangshs@sysucc.org.cn), Tel : 86-20-87342282;

Miao Chen, Sun Yat-sen University Cancer Center, Guangzhou, China, E-mail: chenmaio@sysucc.org.cn, Tel : 86-20-87342300.

**This PDF file includes:**

[Materials and Methods](#methods)

[Figures. S1 to S6](#figure)

[Table S1 to S7](#tables)

Supplementary ChIP seq data (excel)

**Methods and materials**

**1 Cell lines and cell culture**

All breast cancer (BrC) cell lines including SUM-159PT, MDA-MB-231, BT-549, SKBR-3, BT-474, MCF-7, T47D and HEK293T were obtained from American Type Culture Collection (ATCC, Manassas, VA). BT-549 and MCF-7 were cultured in RPMI-1640 (Gibco Invitrogen, Carlsbad, CA) medium containing 10% fetal bovine serum (FBS), and the others were cultured in DMEM (Gibco Invitrogen, Carlsbad, CA) medium containing 10% FBS at 37 °C and 5% CO_2_.

**2 Transient transfection and lentivirus**

Small interfering RNAs (siRNAs) were purchased from Ribobio (Guangzhou, China). Control siRNA and siCPSF4 were transfected with Lipofectamine 3000 (Invitrogen, USA). The oligonucleotides for shCPSF4 and siCPSF4 are listed in Table S5. A series of assays were performed after 48 hours of transfection. The knockdown efficiency was detected by qRT-PCR within 48 hours after transfection, western blot analysis within 72 hours after transfection.

Lentivirus encoding CPSF4 was purchased from GenePharma (Suzhou, China). Plasmids encoding short hairpin RNAs (shRNAs) against CPSF4 were purchased from GeneCopoeia (Guangzhou, China). The full-length cDNA of human MDM4 was cloned into the pSin vector. To obtain stable expression cell lines, transfected cells were treated with puromycin for 2 weeks. Knockdown or overexpression was confirmed by western blotting.

**3 RNA extraction and** **quantitative RT-PCR**

We extracted total RNA from cultured cancer cells by using the RaPure Total RNA Micro Kit (Magen, Guangzhou, China). RNAs were reverse transcribed using the HiScript Q RT SuperMix for qPCR (Vazyme Nanjing, China). Beta-actin was used as an internal control. Quantitative real-time PCR (qRT-PCR) was performed by using ChamQ SYBR qPCR Green Master Mix (Vazyme Nanjing, China).

The mRNA of MDM4-S was detected by qRT-PCR as described by Kristiaan Lenos et.al, other qRT-PCR assays were performed according to the manufacturer’s protocol. Fold changes were calculated by relative quantification (2^–ΔΔCt^). The primers are described in Table S4.

**4 Western blotting**

Total protein from cultured cells was collected and separated on a 10%-12% SDS/PAGE gel and transferred onto PVDF membranes. Membranes were blocked by 5% defatted milk (BD Difco Skim milk, France) and then incubated with the primary antibodies and the horseradish peroxidase (HRP)-conjugated secondary antibody. Finally, the protein bands were detected by using an electrochemiluminescence (ECL) system.

The primary antibodies, except for beta-actin, were diluted at 1:1000 respectively for western blotting. Beta-actin was diluted at 1:2000 for western blotting. The second antibody was diluted at 1:10,000. Transfection effects were detected 72 hours after transfection. The antibodies used in this study are listed in Table S6.

**5 Migration and invasion assays**

Transwell assays were performed by using 24-well transwell plates with an 8μm pore size (Corning, USA). For invasion assay, the membrane was coated with 30μl of 1:12 diluted matrigel (BD Biosciences, USA) and incubated at 37℃ overnight. Then, 5-7.5×10^4^ cells were resuspended in 200μl serum-free DMEM and then added into the cell culture inserts. For migration assay, 5×10^4^ cells with 200μl serum-free DMEM were added into cell culture inserts without the matrigel. The bottom chambers were all filled with 600μl 20% FBS DMEM medium. The plates were incubated at 37℃ in 5% CO_2_ for 24 hours, and then fixed in formaldehyde and stained with crystal violet for 15 min. Final images were taken by microscope at 200× magnification. For the wound scratch assay, we used a 200μl sterile pipette tip to create a wound gap, and then incubated for 24 hours with the DMEM medium without FBS. Images were photographed by microscope at 0, 15, 24 hours.

**6 Cell counting kit-8 assay**

We transfected cells with siNC or siRNA, respectively. Subsequent cells were seeded at 2×10^3^ cells per well in 96-well plates at 24h after transfection. Cells were treated with DMEM with 10%FBS and incubated at 37℃. Cell proliferation was measured by CCK8 (GLPBIO, CA USA). We detected absorbance with a wavelength of 450nm at 72h after seeding plates.

**7 Chromatin immunoprecipitation (ChIP) assay, ChIP-qPCR and ChIP-seq**

We performed ChIP assay by using the ChIP Kit (Millipore and Cell Signaling Technology). The protocol is briefly described as follows: MDA-MB-231-CPSF4 cells, which overexpressed CPSF4 and cultured in 150mm culture dish, were fixed with 1% formaldehyde, digested by micrococcal nuclease, and sonicated to generate DNA fragments of 100–1000 bp. Then, the cell lysate was divided into three groups as follows: input, negative control (IgG), and ChIP groups. IgG and ChIP groups were used for immunoprecipitation overnight with anti-IgG or anti-CPSF4 antibody, respectively, and then immunoprecipitated with protein A/protein G magnetic beads. Finally, DNA samples for qPCR were reverse cross-linked and purified. qPCR was performed as described previously. The amplified products were separated by 2% agarose gel with golden view staining (Gold View I, USA). Images were taken under ultraviolet light. The primers are listed in Table S4. The primary antibodies are listed in Table S6.

The quality of ChIP-sequencing (ChIP-seq) samples, including input and ChIP groups, was strictly controlled by the sequencing company. DNA samples were amplified using nonbiased conditions and then sequenced via HiSeq 2500. The remaining sequences were referred to the human reference genome (GRGH37, hg19). The visual sequencing data were shown by using Integrative Genomics Viewer (IGV 2.4.5). Biological process analysis of the promoter cluster was conducted by using the Database for Annotation, Visualization and Integrated Discovery (DAVID, <https://david.ncifcrf.gov/>). The ChIP-seq analysis data were shown in supplementary data.

**8 Dual-luciferase reporter assay**

The promoter region of MDM4 was cloned into the pGL4.10 vector, including a 1541 bp full-length fragment (-1294~+247 to TSS) and a 312bp fragment (-65~+247 to TSS) from the MDM4 promoter. Moreover, we conducted MDM4 promoter luciferase reporter plasmids which deleted △A (a possible binding region of CPSF4 identified by ChIP-seq, +178~+215 to TSS), △1 (+178~+187 to TSS), △2 (+188~+197 to TSS), △3 (+198~+207 to TSS), and △4 (+208~+215 to TSS) region, respectively. MDA-MB-231 cells were plated on 24-well plates and co-transfected with CPSF4 siRNA, MDM4 promoter-Luci plasmids and renilla luciferase plasmids. Lipofectamine 3000 (Invitrogen, USA) was used during the transfection. Then the 24-well plates were incubated at 37℃. We analyzed the expression of firefly and renilla luciferases activities 72h after transfection by using the Dual-Luciferases Reporter Assay kit (E1910, Promega) and Promega GLOMAX. The pGL4.10 vector was used as negative control.

**9 mRNA and protein stability assay**

We treated MDA-MB-231 cells with Actinomycin D (0.5ug/ml, Abmole, Houston USA) at 2, 4, 6, 8h respectively before collecting RNA. Total RNA was collected at 48h following siNC or siCPSF4 transfection, respectively. Then a qRT-PCR was performed to examine the expression of MDM4. The primers were listed in Table S4.

Cycloheximide (100uM, Yeasen, Shanghai China) was also used to treat MDA-MB-231 cells, which were transfected with CPSF4 overexpression lentivirus. We collected the cell protein at 4, 6, 8, 10h after treatment and performed western blotting.

**10 Animal experiments**

Stable cell lines were established by transfecting specific lentivirus and selected by puromycin. The expression of CPSF4 or MDM4 after silencing or overexpressing was tested by western blotting and IHC staining. Four-week specific pathogen-free (SPF)-grade nude mice were purchased from Beijing Vital River Laboratory Animal Technology Company and kept in the Animal Center at Sun Yat-Sen University. Next, we conducted tail-vein lung metastasis animal models by injecting 5x106 cells suspended in 100μl 1× phosphate buffer saline (PBS). After 12 weeks, the nude mice were sacrificed, and their lungs were separated for further detection. We fixed the separated lungs in formaldehyde and then embedded, sectioned, and stained with hematoxylin and eosin (H&E), which is an immunohistochemistry (IHC)-specific staining. The number of metastatic modules was counted and analyzed. Animal experiments were conducted in accordance with the process approved by the Ethics Committees of Sun Yat-Sen University Cancer Center.

**11 Tissue microarrays, human tissue specimens and IHC staining**

A total of 129 samples were enrolled for IHC staining, of which 101 samples within a tissue microarray were purchased from Outdo Biotech Co., Ltd. (Shanghai, China), and 28 were from Sun Yat-Sen University Cancer Center. The primary antibodies were diluted at 1:100 for IHC staining. For color development, 3,3'-diaminobenzidine (DAB; Dako) staining was used. The primary antibodies are listed in Table S7.

The staining intensity of CPSF4 and MDM4 were classified into the following four classes: 0 for no staining, 1 for weak staining, 2 for moderate staining, and 3 for strong staining. Percentages of stained cells of <25%, 25%–50%, 51%–75% and >75% were defined as 0, 1, 2, 3, and 4, respectively. The scores were calculated as the staining intensity value multiplied by the staining percentage value. When the staining score was ≥7, the expression of tissue protein was defined as a high level. In contrast, when the staining score was ≤6, the expression of tissue protein was considered low level.

**12 Statistical analysis**

Cell numbers, wound gap areas, and western blotting were analyzed by Image-pro-plus (version 6.0). The IBM SPSS statistical software (version 20.0), Stata (version 16.0), and GraphPad 7.0 were used for statistical analysis. Continuous data were presented as the mean±SD and categorical data were presented as percentages. Chi-squared test and Student’s t-test were applied for the significant difference analysis where applicable. Kaplan-Meier analysis was used for survival analysis and compared with the log-rank test. Hazard ratio (HR) and corresponding 95% confidence interval (CI) was determined through Cox proportional hazards regression analysis. Multivariate analysis was used to identify independent prognostic factors associated with overall survival. The performance for predicting survival was evaluated by calculating the concordance index (C-index). A larger C-index indicated more accurate prognostic stratification. Statistically significant differences were considered when *P* values were <0.05.

**Figure S1**


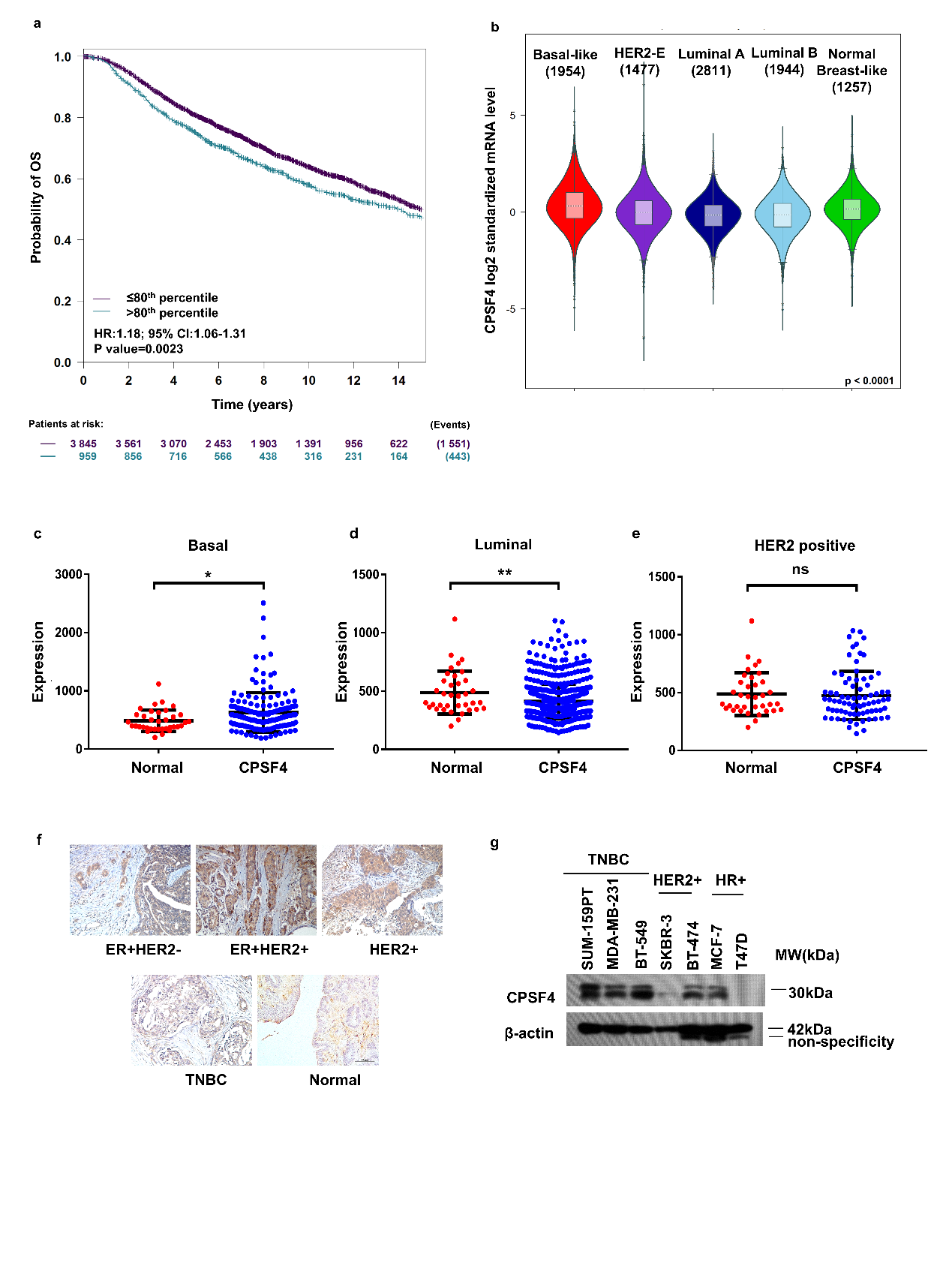


**Figure S1. Expression of CPSF4 elevated in breast cancer. a** Prognostic value of different CPSF4 expression level in breast cancer in the GenExMiner database. **b** Expression analysis of CPSF4 in various breast cancer subtypes in the GenExMiner database. **c-e** Expression of CPSF4 in **(c)** basal-like, **(d)** luminal, and **(e)** HER2-positive tumors in the TCGA database. **f** The representative images of IHC staining in BrC and normal breast tissues. **g** The baseline expression of CPSF4 in BrC cell lines. **P*<0.05, ***P*<0.01.

**Figure S2**

**
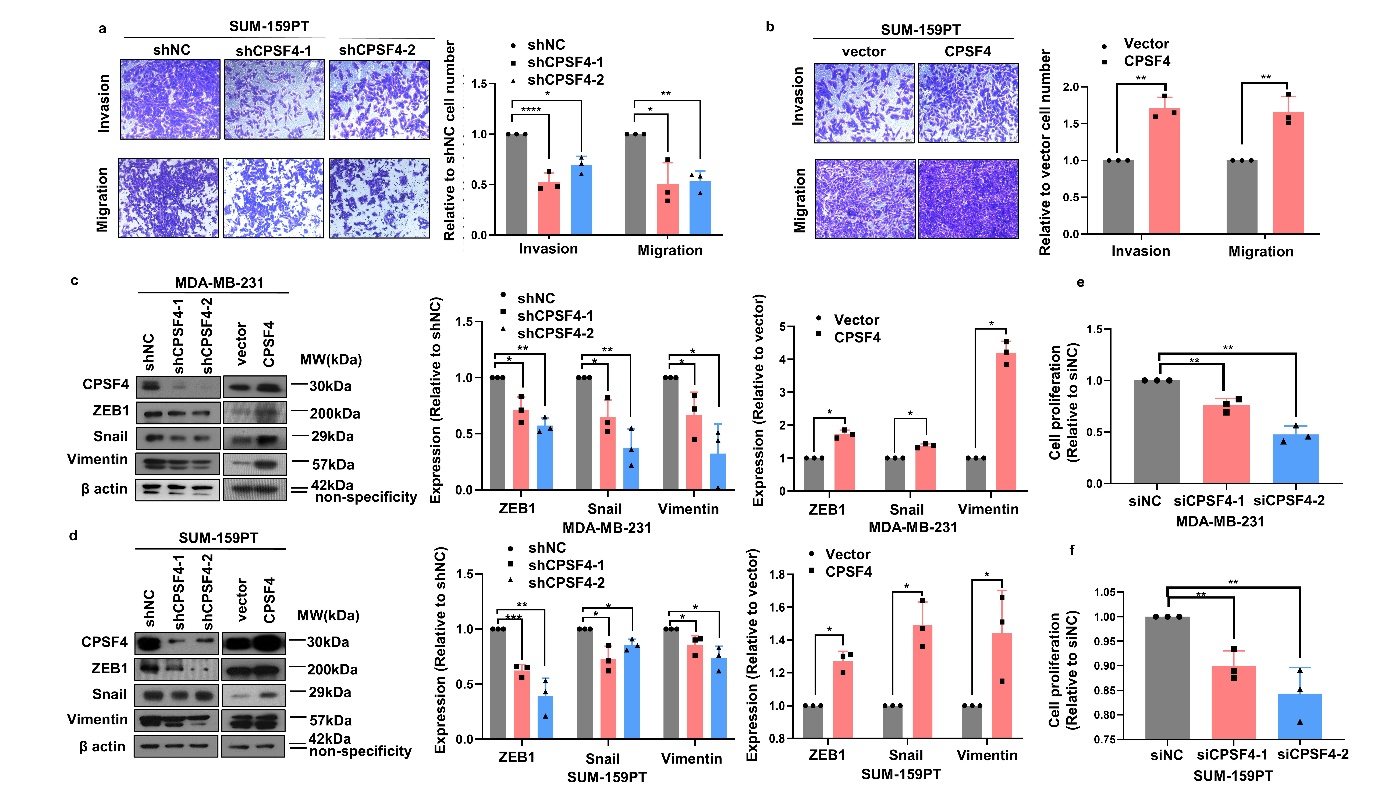
**

**Figure S2. CPSF4 promotes cell invasion, migration and proliferation in TNBC. a, b** SUM-159PT cells were transfected with shNC, shRNA-CPSF4 **(a)**, and CPSF4-overexpression lentivirus **(b)**, respectively. All above cells were used to perform transwell migration and matrigel invasion assays. **c, d** Protein expression of EMT-related markers were detected by western blotting. Band intensity analysis was also performed. **e,f** MDA-MB-231 **(e)** and SUM-159PT **(f)** cells were transfected with siNC, siCPSF4, respectively. All above cells were used to perform CCK8 assay. **P*<0.05, ***P*<0.01, *** *P*<0.001, and *****P*<0.0001.

**Figure S3**

**
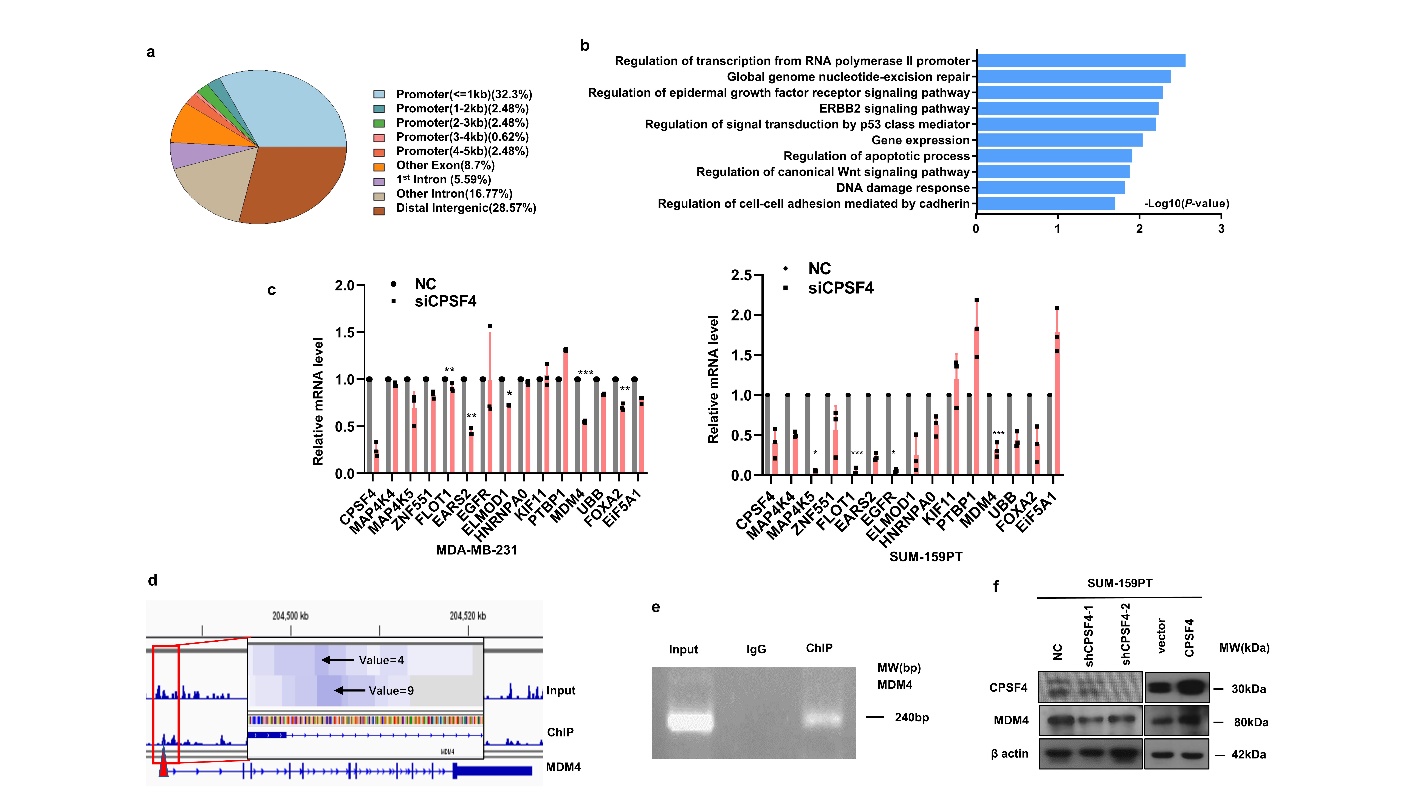
**

**Figure S3. CPSF4 transcriptionally regulates MDM4. a** The target gene landscape identified by ChIP-seq. **b** KEGG pathway analysis of target genes. **c** Target genes verified by qRT-PCR after silencing CPSF4 in MDA-MB-231 and SUM-159PT cells. **d** The ChIP-seq track of CPSF4 at MDM4 promoter (The red triangle indicated the location of CPSF4). **e** ChIP-qPCR agarose gel electrophoresis analysis. **f** Western blotting performed to detect the expression of MDM4 after silencing or overexpressing CPSF4 in SUM-159PT cells. **P*<0.05, ***P*<0.01, *** *P*<0.001, and *****P*<0.0001.

**Figure S4**

**
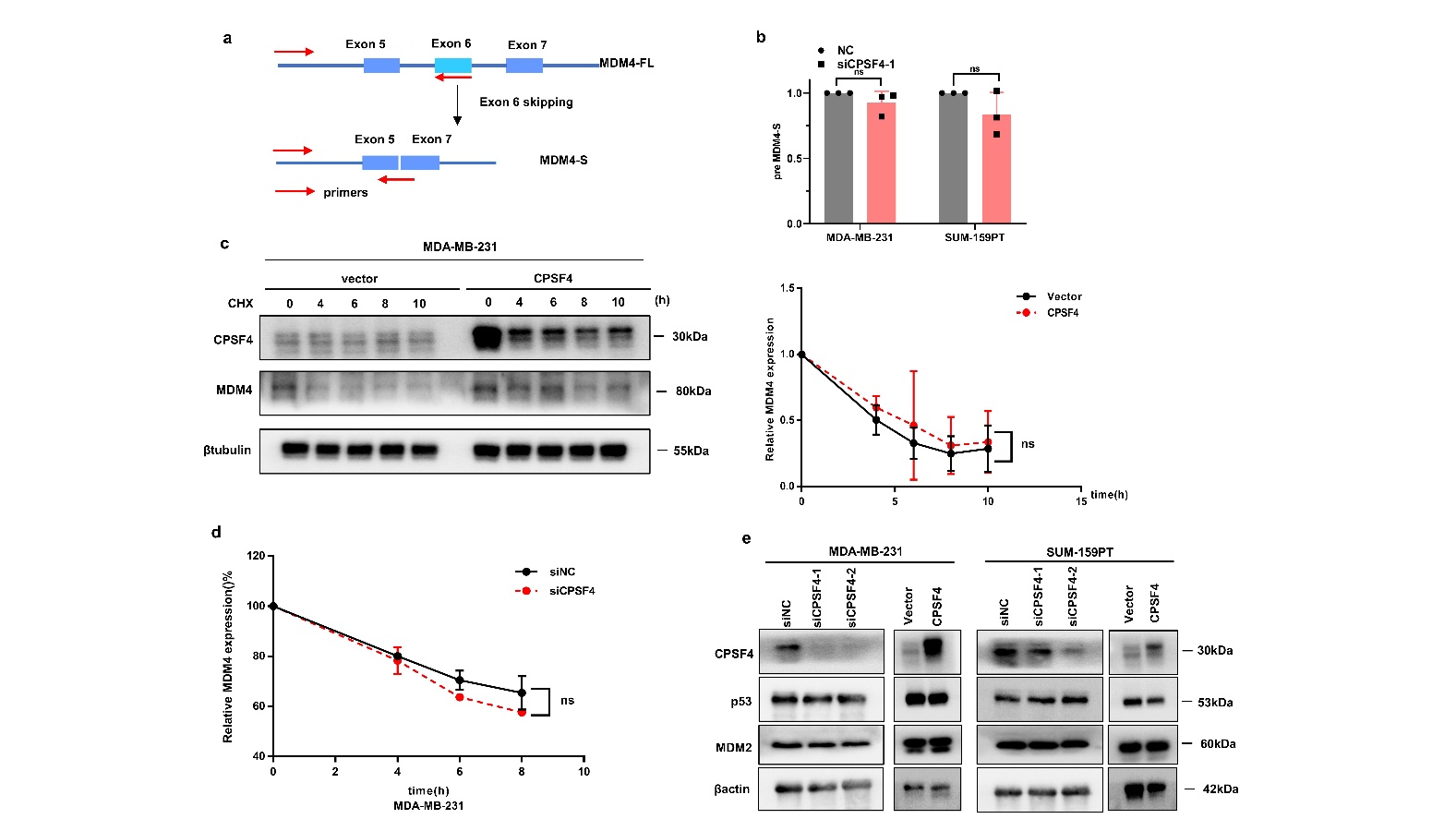
**

**Figure S4. CPSF4 do not regulate alternative splicing, RNA stability and protein stability process of MDM4. a** Schematic diagram of alternative splicing of MDM4. **b** The expression of MDM4-S detected by qRT-PCR. **c** Western blot analyses of CPSF4-overexpression stable cells that were treated with Cycloheximide (100uM) at 4, 6, 8, 10h before collecting protein. **d** qRT-PCR analyses of MDA-MB-231 cells that were treated with Actinomycin D (0.5ug/ml) at 2, 4, 6, 8h before collecting RNA. Total RNA was collected at 48h following siNC or siCPSF4 transfection, respectively. **e** Western blot analyses of p53 and MDM2 expression after silencing or overexpressing CPSF4, respectively. ns: No significant difference.

**Figure S5**

**
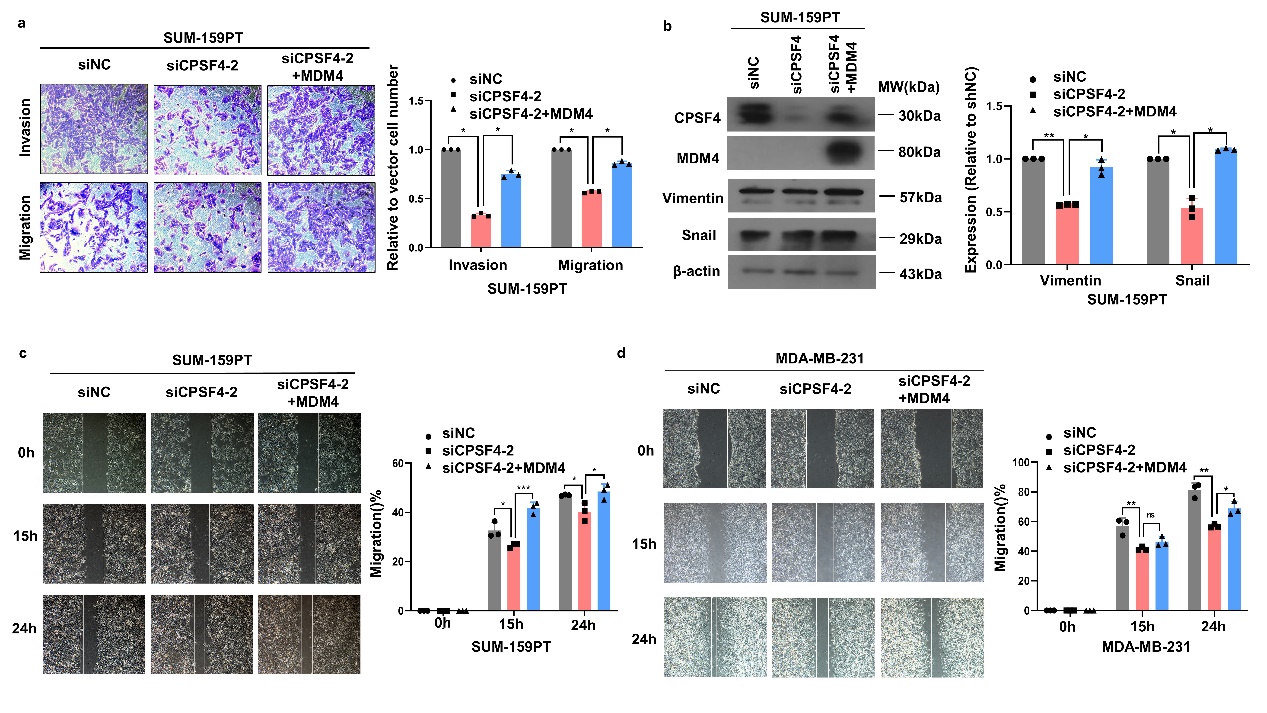
**

**Figure S5. CPSF4 regulates TNBC cell metastasis partly through MDM4 in vitro. a** Transwell migration and matrigel invasion rescue assays performed in CPSF4-silencing SUM-159PT TNBC cells that were transfected with or without MDM4-overexpressing lentivirus. **b** Expression of EMT-related markers detected by western blotting and analyzed by image-pro-plus in CPSF4-silencing SUM-159PT TNBC cells that were transfected with or without MDM4-overexpressing lentivirus. **c, d** Wound scratch rescue assays performed in CPSF4-silencing **(c)** SUM-159PT and **(d)** MDA-MB-231 TNBC cells that were transfected with or without MDM4-overexpressing lentivirus. ns: No significant difference, **P*<0.05, ***P*<0.01, and ****P*<0.001.

**Figure S6**

**
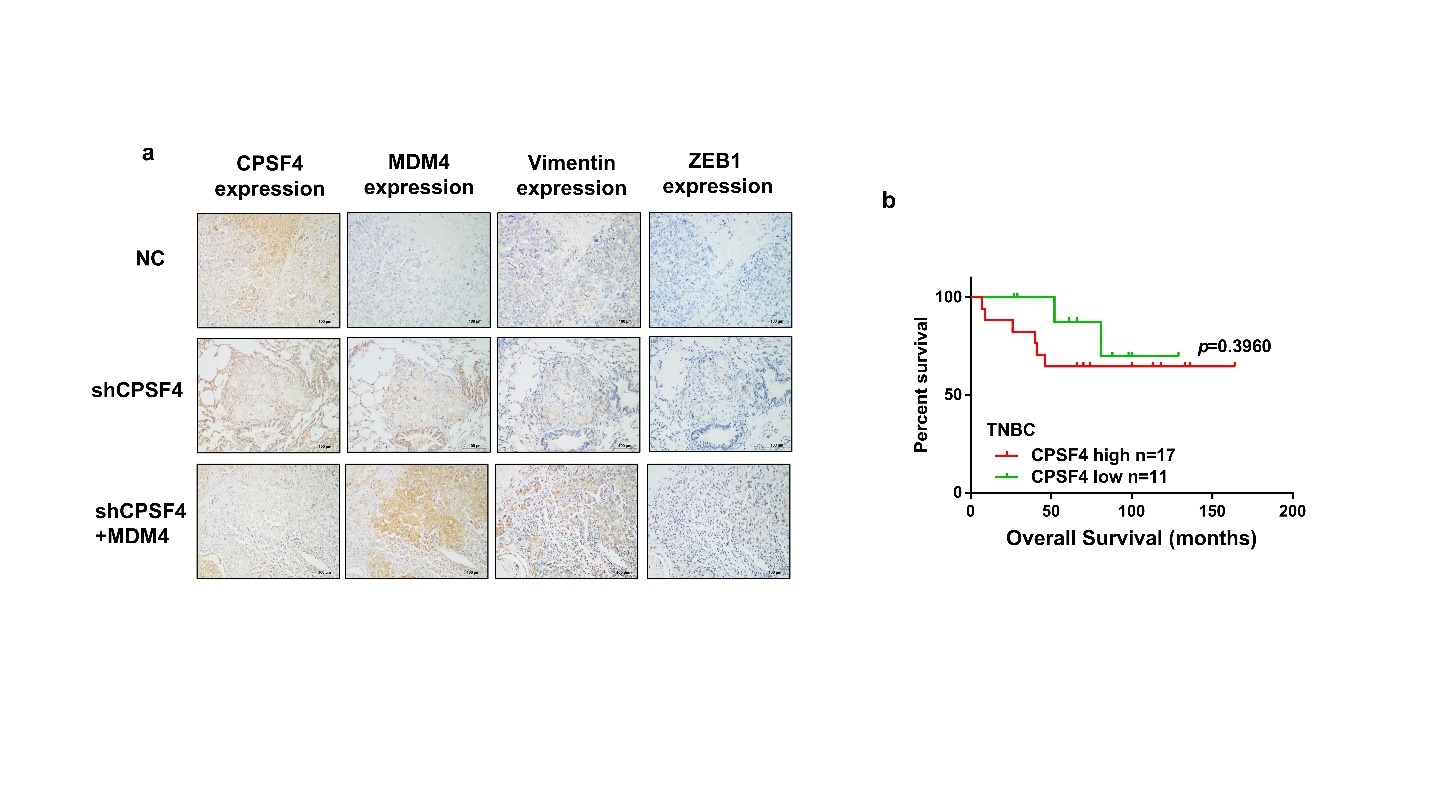
**

**Figure S6. Elevated expression of CPSF4 and MDM4 are related to poor outcomes of patients with BrC. a** MDA-MB-231 cells were transfected with shRNA-CPSF4 or MDM4-overexpressing lentivirus. These cells were injected into the tail-vein of 4-week-old nude mice to establish metastatic models. Representative images of IHC staining of CPSF4, MDM4, vimentin and ZEB1. **b** The Kaplan-Meier curve of overall survival related to high or low expression of CPSF4 in TNBC (*P*=0.3960).

**Table S1 Multivariate analysis of prognostic factors for OS in TNBC population.**

| **TNBC subgroup** | **No. of patients** | **HR (95% CI)** | **P value** |
| --- | --- | --- | --- |
| Age, years | 28 | 0.92 (0.83-1.02) | 0.132 |
| T stage |  |  | 0.334 |
| T1 | 5 | 1.00 (Reference) |  |
| T2/3 | 23 | 3.36 (0.29-39.16) |  |
| Node status |  |  | 0.55 |
| Negative | 13 | 1.00 (Reference) |  |
| Positive | 15 | 1.84 (0.25-13.53) |  |
| TNM stage |  |  | 0.083 |
| Stage I/II | 22 | 1.00 (Reference) |  |
| Stage III | 6 | 5.78 (0.80-41.97) |  |
| CPSF4-MDM4 level * |  |  | 0.006 |
| Low | 19 | 1.00 (Reference) |  |
| High | 9 | 14.22 (2.11-95.88) |  |

* CPSF4-MDM4 level is defined as high when both CPSF4 and MDM4 level were high, and low when either CPSF4 or MDM4 level was low.

**Table S2. Multivariate analysis of prognostic factors for OS in overall BrC population.**

| **Total population** | **No. of patients** | **HR (95% CI)** | **P value** |
| --- | --- | --- | --- |
| Age, years | 129 | 1.04 (1.00-1.08) | 0.039 |
| T stage |  |  | 0.164 |
| T1 | 46 | 1.00 (Reference) |  |
| T2/3 | 83 | 2.05 (0.75-5.63) |  |
| Node status |  |  | 0.03 |
| Negative | 65 | 1.00 (Reference) |  |
| Positive | 64 | 3.66 (1.14-11.80) |  |
| TNM stage |  |  | 0.525 |
| Stage I/II | 90 | 1.00 (Reference) |  |
| Stage III | 39 | 1.38 (0.51-3.76) |  |
| CPSF4-MDM4 level * |  |  | 0.011 |
| Low | 85 | 1.00 (Reference) |  |
| High | 44 | 3.01 (1.28-7.08) |  |

* CPSF4-MDM4 level is defined as high when both CPSF4 and MDM4 level were high, and low when either CPSF4 or MDM4 level was low.

**Table S3. The C-index of different multivariate Cox regression models.**

| **Population** | **C-index (95% CI)** |
| --- | --- |
| TNBC population |  |
| Clinical factors only | 0.726 (0.504-0.948) |
| Clinical factors + CPSF4-MDM4 level | 0.833 (0.647-1.02) |
| Total population |  |
| Clinical factors only | 0.583 (0.336-0.831) |
| Clinical factors + CPSF4-MDM4 level | 0.685 (0.460-0.909) |

Clinical factors: including age, T stage, N stage, and TNM stage.

**Table S4 Primers used in study.**

| CPSF4 forward | 5′-TGGCCTATGCAAGAAAGGGG-3′ |
| --- | --- |
| CPSF4 reverse | 5′-TGCATCTGTTGGCGTAGTGT-3′ |
| GAPDH forward | 5′-TGTGGGCATCAATGGATTTGG-3′ |
| GAPDH reverse | 5′-ACACCATGTATTCCGGGTCAAT-3′ |
| MAP4K4 forward | 5′-GACTCCCCTGCAAAAAGTCTG-3′ |
| MAP4K4 reverse | 5′-GTCCATAGGTGCCATTTCCAA-3′ |
| MAP4K5 forward | 5′-CCCCATGCAATCATTCGTCAT-3′ |
| MAP4K5 reverse | 5′-CCCATTTCATCTCGTGCTTCTG-3′ |
| ZNF551 forward | 5′-TCGGCTCAGGGTATGACCTTT-3′ |
| ZNF551 reverse | 5′-ATCGCAGTACAGGAACCTCTG-3′ |
| FLOT1 forward | 5′-GCCCTGCATCCAACAGATCC-3′ |
| FLOT1 reverse | 5′-AATGCCAGTGACTGAGATGGG-3′ |
| EARS2 forward | 5′-CAAGAGGCAAGGGGACGTTTT-3′ |
| EARS2 reverse | 5′-GTGTCAGGTTGAACTGTGTGA-3′ |
| EGFR forward | 5′-TTGCCGCAAAGTGTGTAACG-3′ |
| EGFR reverse | 5′-GTCACCCCTAAATGCCACCG-3′ |
| ELMOD1 forward | 5′-GTGTTCACCCCGACGCTATT-3′ |
| ELMOD1 reverse | 5′-AGCTGTGGATTTACGTCAGGAT-3′ |
| HNRNPA0 forward | 5′-GCGACCTGATCGAGCACTTC-3′ |
| HNRNPA0 reverse | 5′-CGCGTCGTGATTCTGGAAATAC-3′ |
| KIF11 forward | 5′-AGCAAGCTGCTTAACACAGTT-3′ |
| KIF11 reverse | 5′-CCTTCTTACGATCCAGTTTGGAA-3′ |
| PTBP1 forward | 5′-AGCGCGTGAAGATCCTGTTC-3′ |
| PTBP1 reverse | 5′-CAGGGGTGAGTTGCCGTAG-3′ |
| MDM4 forward | 5′-TGATTGTCGAAGAACCATTTCGG-3′ |
| MDM4 reverse | 5′-TGCAGGGATCAAAAAGTTTGGAG-3′ |
| RB1CC1 forward | 5′-GAAAGAGCTTGCTCAGGGATT-3′ |
| RB1CC1 reverse | 5′-TCATCAACTGATTTGCGTGACT-3′ |
| UBB forward | 5′-GGTCCTGCGTCTGAGAGGT-3′ |
| UBB reverse | 5′-GGCCTTCACATTTTCGATGGT-3′ |
| FOXA2 forward | 5′-GGAGCAGCTACTATGCAGAGC-3′ |
| FOXA2 reverse | 5′-CGTGTTCATGCCGTTCATCC-3′ |
| eIF5AI forward | 5′-GGACTTCGAGACAGGAGATGC-3′ |
| eIF5AI reverse | 5′-TCATTCCTTTTGATGTTGGGGAC-3′ |
| MDM4 promoter forward (ChIP-qPCR) | 5′-CGGAAGTTGCGGCTTCATTA-3′ |
| MDM4 promoter reverse (ChIP-qPCR) | 5′-CCCGACCCACCTCTCAAG-3′ |
| MDM4-FL forward | 5′-CAGCAGGTGCGCAAGGTGAA-3′ |
| MDM4-FL reverse | 5′-CTGTGCGAGAGCGAGAGTCTG-3′ |
| MDM4-S forward | 5′-CAGCAGGTGCGCAAGGTGAA-3′ |
| MDM4-S reverse | 5′-GCACTTTGCTGTAGTAGCAGTG-3′ |

**Table S5 siRNA used in study**

| shCPSF4-1 and siCPSF4-1 | 5’-GAGTCATCTGTGTGAATTA-3’ |
| --- | --- |
| shCPSF4-2 and siCPSF4-2 | 5’-CATGCACCCTCGATTTGAA-3’ |

**Table S6 Antibodies for western blotting and ChIP.**

| **Antibody** | **Company** | **Cat No.** |
| --- | --- | --- |
| CPSF4 | Novus, USA | NB100-79827 |
| MDM4 | Proteintech, China | 17914-1-AP |
| MDM2 | Proteintech, China | 19058-1-AP |
| P53 | Proteintech, China | 10442-1-AP |
| IgG | Cell signaling technology, USA | 2729 |
| Snail (C15D3) | Cell signaling technology, USA | 9782 |
| Vimentin (D21H3) | Cell signaling technology, USA | [9782](https://www.cst-c.com.cn/products/primary-antibodies/epithelial-mesenchymal-transition-emt-antibody-sampler-kit/9782?site-search-type=Products) |
| ZEB1 (D80D3) | Cell signaling technology, USA | [9782](https://www.cst-c.com.cn/products/primary-antibodies/epithelial-mesenchymal-transition-emt-antibody-sampler-kit/9782?site-search-type=Products) |
| Beta-actin | Proteintech, China | 20536-1-AP |
| Beta-tubulin | Proteintech, China | 66240-1-IP |
| HRP-labeled Goat Anti-Rabbit IgG(H+L) | Promega, USA | W4018 |

**Table S7 Antibodies for immunohistochemistry.**

| **Antibody** | **Company** | **Cat No.** |
| --- | --- | --- |
| CPSF4 | Proteintech, China | 15023-1-AP |
| MDM4 | Proteintech, China | 17914-1-AP |
| Vimentin | Servicebio, China | GB14167 |
| ZEB1 | Servicebio, China | GB11513 |
